# Comparison of the 5-Year Outcomes Between Standard and Short Fit-and-Fill Stems in Japanese Populations

**DOI:** 10.1016/j.artd.2022.03.023

**Published:** 2022-04-25

**Authors:** Suguru Kato, Masahiko Nozawa, Sungon Kim, Yuko Sakamoto, Hironori Ochi, Muneaki Ishijima

**Affiliations:** a Department of Orthopaedic Surgery, Juntendo University Nerima Hospital, Tokyo, Japan; b Department of Orthopaedic Surgery, Juntendo University, Tokyo, Japan

**Keywords:** Total hip arthroplasty, Fit-and-fill stems, Cementless stem, Stress shielding, Japanese Orthopedic Association score

## Abstract

**Background:**

Fit-and-fill stems are known to have excellent outcomes; however, severe stress shielding has been reported in Japanese populations. Short fit-and-fill stems were modified for Asians; however, there have been no previous reports of its outcome. In this study, we compared the 5-year (mean 68-month) outcomes of 2 fit-and-fill stems with different lengths (standard or short).

**Material and methods:**

We reviewed 100 total hip arthroplasties in each standard- or short-stem group. Radiographs were evaluated for femoral morphology, stress shielding, bone remodeling, and fixation. Clinical evaluation was performed using the Japanese Orthopaedic Association (JOA) scores.

**Results:**

There was no difference in the degree of stress shielding between the 2 groups. Significant differences were observed in radiolucent lines in zone 4 (*P* = .005) and cortical hypertrophy in zone 3 (*P* < .0001) and 5 (*P* = .048) between the 2 groups. The canal flare index (*P* < .0003), cortical index (*P* < .0003), height (*P* < .0345), and stem size (*P* < .0081) individually affected stress shielding in the standard-stem group. In contrast, the cortical index (*P* < .0107) was the only relative factor in the short-stem group. All stems were judged to have bone ingrowth. The JOA score improved significantly (*P* < .0001); however, there were no significant differences between the 2 groups.

**Conclusion:**

The outcomes of both standard and short fit-and-fill stems were favorable. There were no significant differences in the stress shielding or JOA scores. Although there were a few differences in bone remodeling and factors affecting stress shielding, stem length reduction has been achieved without adverse effects with the Japanese femur.

## Introduction

Cementless stems have been widely used in total hip arthroplasty (THA) for decades, and their long-term results have been successful [[1], [2], [3]]. In Japan, various foreign-made implants have been introduced and used, and most of them function well. However, there is concern that some of these implants do not match the morphology of the Asian bone due to their smaller skeleton size and greater femoral bowing [4,5].

Fit-and-fill stems are known for their excellent stability and low frequency of thigh pain postoperatively [[6], [7], [8], [9]]. However, severe and progressive stress shielding has been reported in the Japanese population [10,11]. Fit-and-fill stems are used at our institution, and in 2013, we began to utilize the modified shorter model of the same series to conform to the Asian femur. There are no previous reports comparing standard and short fit-and-fill stems, and the benefit of this modification is still unclear. The purpose of this study was to describe the differences between these 2 implants using retrospective radiographic and clinical results in Japanese populations.

## Material and methods

### Implants

**Figure 1:**
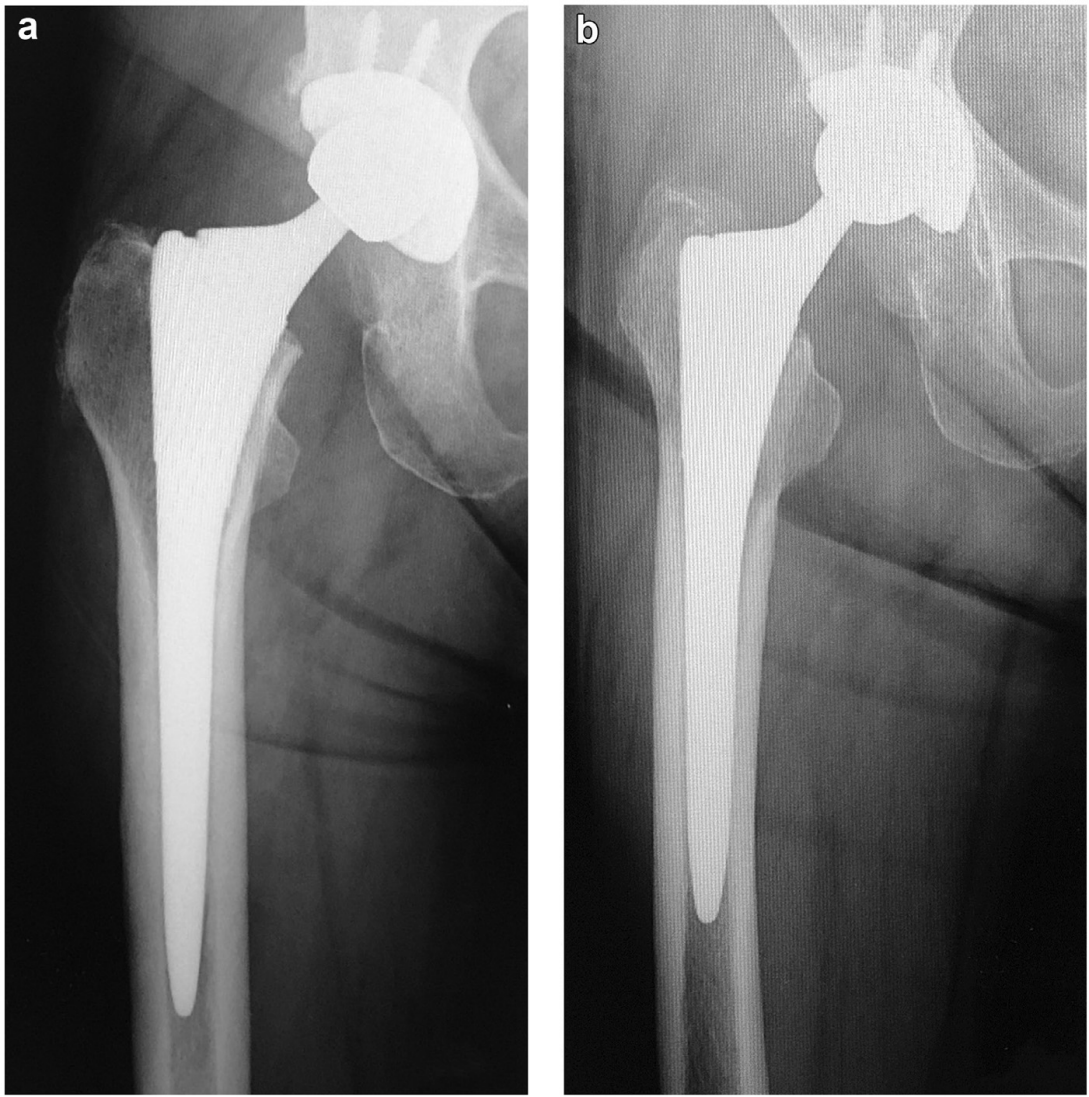
Radiographs of the (a) standard stem and (b) short stem (size 12).

The Synergy (Smith and Nephew, Memphis, TN; hereinafter referred to as “original stem”) stem is a double-tapered metaphyseal filling stem made of a titanium alloy (Ti-6Al-4V). The stem has a rough titanium bead porous coating on the proximal third and is grit-blasted below the proximal third to a point near the end. The distal part of the stem is bullet-shaped and polished. The trunnion diameter is 12/14. The Synergy Select stem (Smith and Nephew, Memphis, TN; hereinafter referred to as “standard stem”) is a minor variation of the same series. The trunnion diameter was changed to 10/12 to maximize the oscillation angle. The stem body design is the same as that of the original stem. The Synergy Select 2 stem (Smith and Nephew, Memphis, TN; hereinafter referred to as “short stem”) is a stem modified to fit the Asian femur. The length of the short stem is 15 mm shorter than that of the standard stem, and the coating and stem proportions are the same. The radiographs of the implants of the same size are shown in Figure 1. All implants were combined with a Reflection (Smith and Nephew, Memphis, TN) cup and OXINIUM (Smith and Nephew, Memphis, TN) head.

### Patients

**Figure 2:**
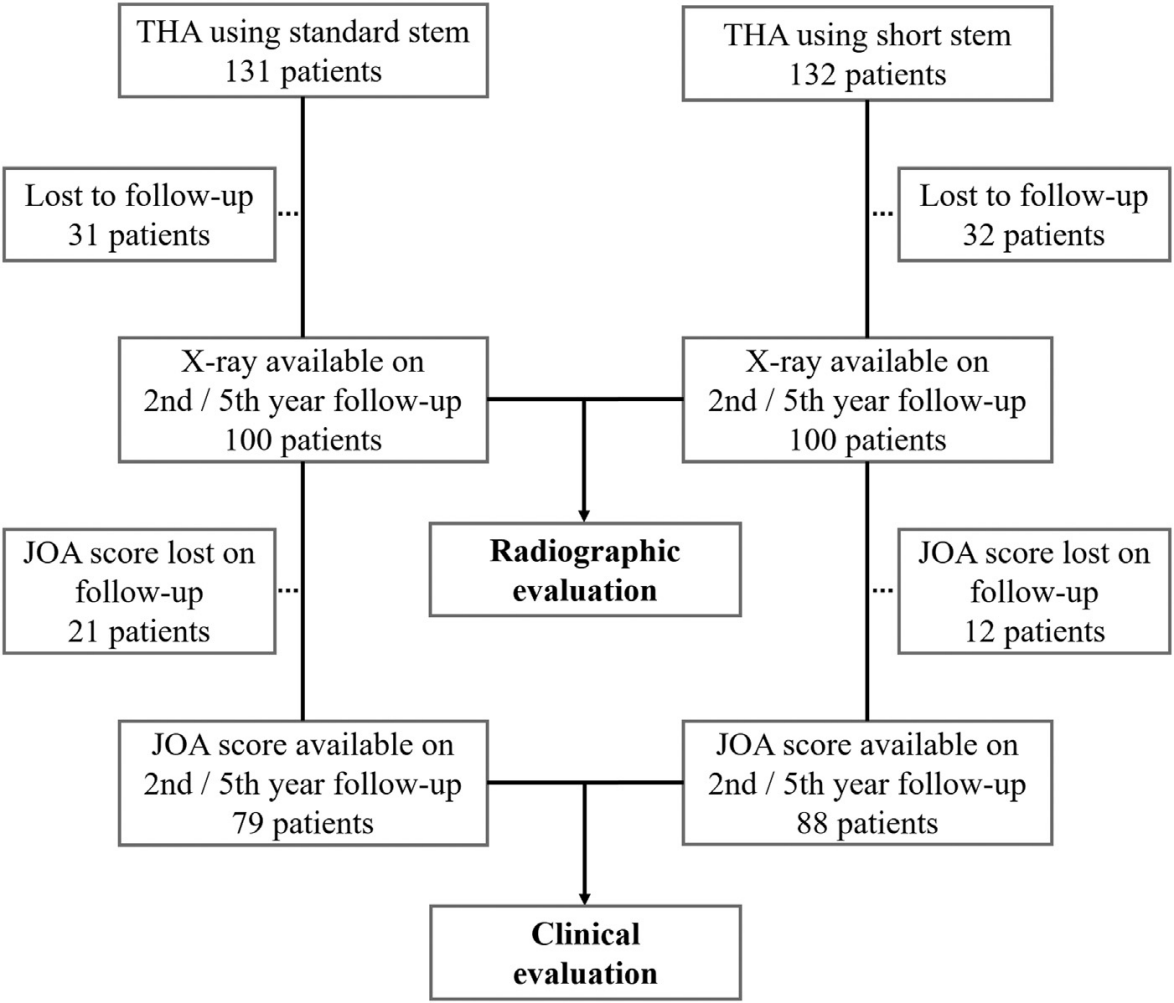
Study flow chart.

We retrospectively reviewed the records of patients who underwent THA at our hospital. Since the standard stem was used until December 2012, we traced 100 hip arthroplasty cases whose radiographs were available at the second (24–35 months) and fifth year (60–71 months) postoperative intervals. A total of 131 THAs with the standard stem were performed between December 2011 and December 2012, with clinical questionnaire scores of 79 cases at the second and fifth year available. The short stem was used from January 2013, and we traced 100 cases that had radiographs available at the second and fifth year postoperative intervals. A total of 132 THAs with the short stem were performed between January 2013 and December 2013, and the clinical questionnaire scores of 88 cases at the second and fifth year were available. The study flowchart is shown in Figure 2. Patients whose records were not available were excluded at each radiographic or clinical evaluation. The protocol of this retrospective study was approved by the institutional review board of our hospital. Informed consent for the study was obtained using the opt-out method via our hospital website.

### Operative procedure

Surgeries were performed using the posterior approach by 8 orthopedic surgeons. The implants were inserted by manual reaming and broaching using standard instruments. All patients were encouraged to ambulate and were allowed full weight-bearing on the first postoperative day. Rehabilitation was performed by physical therapists at the department of rehabilitation in our hospital, according to each patient's progress. Patients were given cefazolin sodium infusion (3 times on the day of operation, 1000 mg each) as a standard antibiotic prophylaxis and subcutaneous injection of enoxaparin sodium (2000IU twice daily for 2 weeks) to prevent thrombosis.

### Radiographic assessments

Anteroposterior radiographs were obtained in the supine position. Radiographic measurements were performed by a single observer (author S.K.) who was not involved in the surgery. The ruler function of the electronic medical record system (HOPE Dr ABLE-GX V01; Fujitsu, Japan) was used for digital analysis.

The femoral morphology was evaluated using the preoperative radiographs. The cortical index (CI) [12], canal-calcar ratio [12], and canal flare index (CFI) [13] were analyzed.

**Figure 3:**
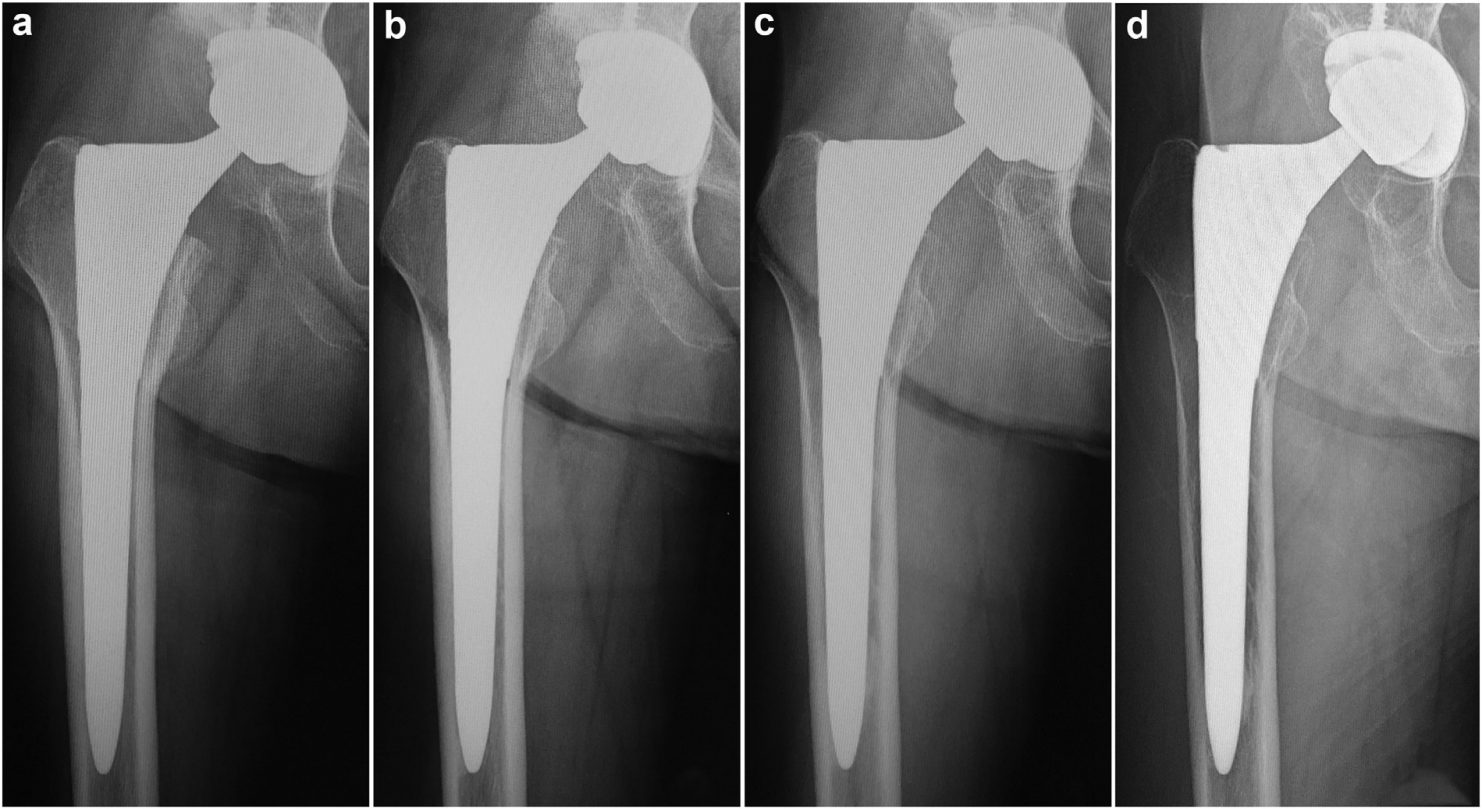
Example of stress shielding observed in standard stem. (a) First degree; (b) second degree; (c) third degree; (d) fourth degree.

**Figure 4:**
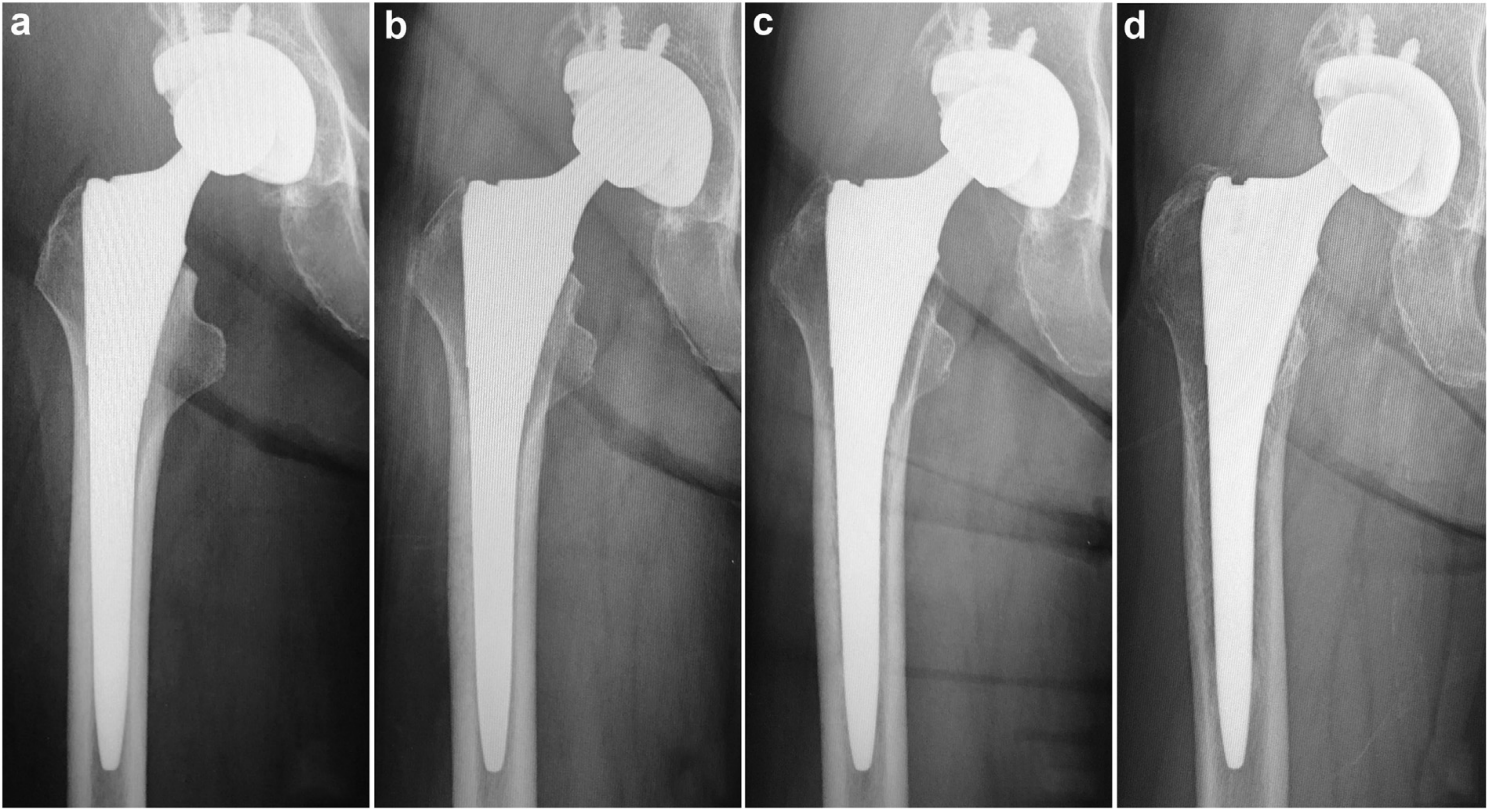
Example of stress shielding observed in short stem. (a) First degree; (b) second degree; (c) third degree; (d) fourth degree.

**Figure 5:**
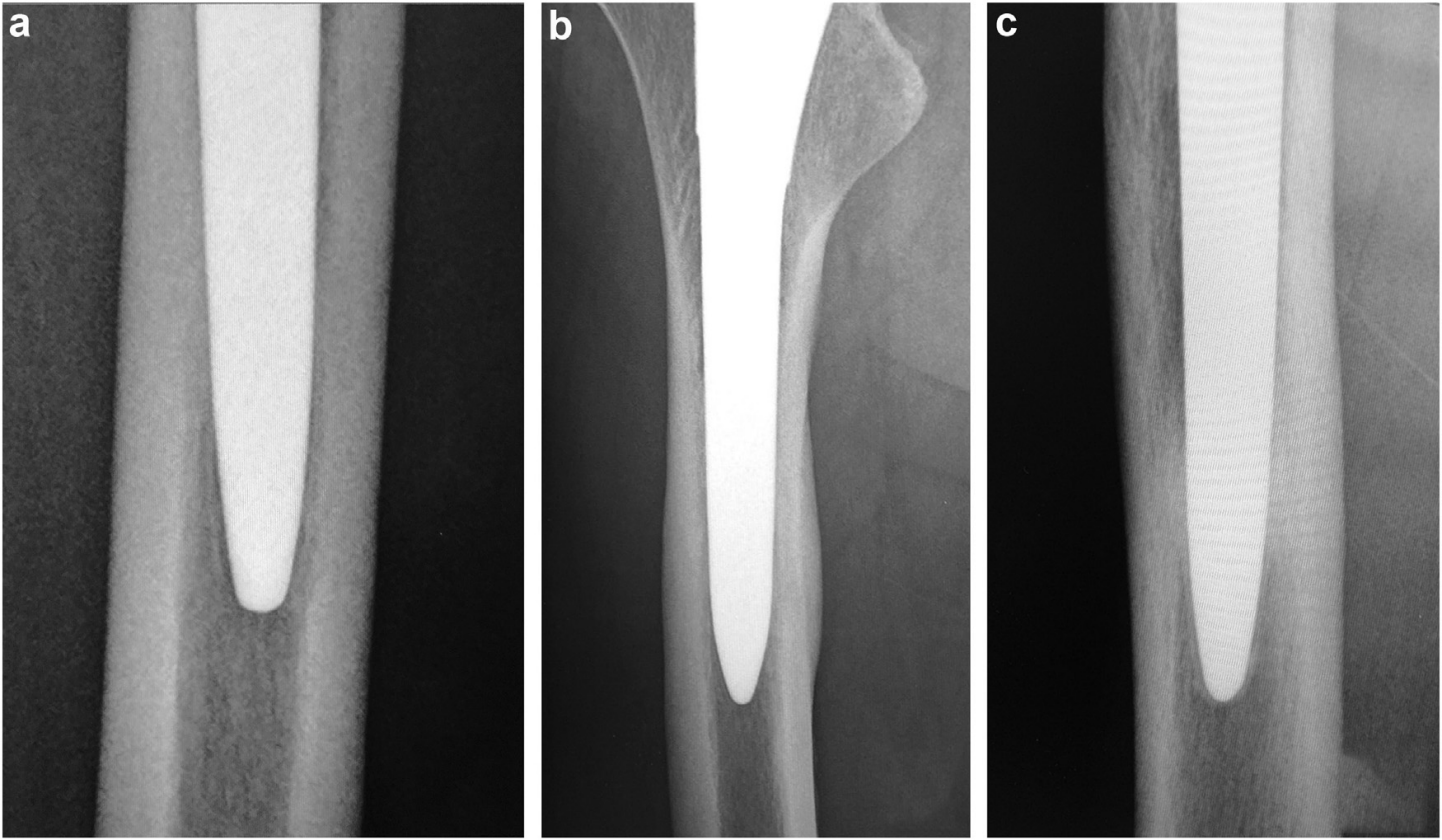
Example of radiological changes observed. (a) Radiolucent line in zone 4; (b) cortical hypertrophy in zone 3 and 5; (c) spot welds in zone 3 and 5.

Radiographs at the second and fifth year were used to evaluate postoperative outcomes. Stress shielding was classified based on Engh's classification [14]. Radiolucent lines, cortical hypertrophy, and spot welds were recorded using the Gruen zones [15]. Typical radiological changes are shown in Figure 3, Figure 4, Figure 5. A stem subsidence greater than 2 mm was considered to be positive. Stem biological fixation was evaluated according to Engh’s criteria [16].

### Clinical evaluation

Japanese Orthopaedic Association (JOA) scores [17] were recorded preoperatively and at the second and fifth years postoperatively. The JOA score consists of 40 points for pain, 20 points for range of motion, 20 points for walking ability, and 20 points for activities of daily living. Thigh pain was considered to be positive when the JOA score for pain was recorded as 30 points (described as “no pain during walking; however, pain at the start of walking or after long-distance walking may occur occasionally”) or less. In addition, complications (dislocations and fractures) found during follow-up were also recorded.

### Statistical analysis

An unpaired t-test was used to compare continuous variables between the standard- and short-stem groups. Paired t-test was used to compare the JOA scores between each follow-up. Fisher's exact test (for a 2 × 2 table) or Chi-square test was used for categorical variables. Stress shielding was analyzed using the Mann-Whitney test to compare the standard- and short-stem groups, while the Wilcoxon test was used to compare each follow-up. Multiple regression analysis was performed by stepwise selection method that included age, height, body mass index, stem alignment, stem size, CI, canal-calcar ratio, and CFI as independent variables and stress shielding as the dependent variable. Data analysis was performed using Prism 7.04 (GraphPad Software Inc., San Diego, CA) with statistical significance set at *P* < .05.

## Results

There were no significant differences in baseline patients’ characteristics between the 2 groups (Table 1). The Wilcoxon test showed significant progression of stress shielding in both groups (*P* < .0001) from the second to the fifth year (Table 2). However, the degree of stress shielding was not significantly different between the 2 groups in the second (*P* = .17) and fifth year (*P* = .53).

**Table 1:** Patients’ characteristics.

Patient characteristics	Standard stem	Short stem	*P* value
Age (y)	64.0 ± 11.6	65.2 ± 11.4	.45
Height (cm)	156.2 ± 7.7	154.9 ± 7.7	.24
BMI	22.8 ± 3.1	23.0 ± 3.6	.73
Gender (n); M:F	13:87	9:91	.50
Operation side (n); R:L	53:47	55:45	.89
Diagnosis (n)			
OA	88	87	
ON	6	10	
RA	6	3	.37
Stem size	12.3 ± 1.5	12.2 ± 1.5	.47
Stem alignment^a^	0.86 ± 0.94	0.97 ± 1.18	.48
Femoral morphology			
Cortical index	0.52 ± 0.06	0.52 ± 0.06	.68
Canal-to-calcar ratio	0.62 ± 0.07	0.64 ± 0.08	.09
Canal flare index	3.54 ± 0.62	3.58 ± 0.64	.67

BMI, body mass index; OA, osteoarthritis; ON, osteonecrosis; RA, rheumatoid arthritis.

a Varus angle.

**Table 2:** Degree of stress shielding analyzed using the Wilcoxon test.

Degree of stress shielding	Standard stem		Short stem	
	Second year	Fifth year	Second year	Fifth year
First	35	20	19	12
Second	53	57	76	66
Third	10	18	4	21
Fourth	2	5	1	1
*P* value		<.0001^a^		<.0001^a^

a Significant progression of stress shielding from the second to fifth year in both groups is observed.

The number of radiolucent lines, cortical hypertrophy, and spot welds are shown in Table 3. Significant differences were observed in radiolucent lines in zone 4 between the 2 groups (*P* = .005) at the second year follow-up. Similarly, there were significant differences in the amount of cortical hypertrophy between the 2 groups in zone 3 at the second (*P* < .0001) and fifth year (*P* < .0001) and in zone 5 at the fifth year (*P* = .048). The presence of any distal bone fixation (cortical hypertrophy or spot welds in zone 3 or 5) had a significant relationship with severe (third or fourth degree) stress shielding in both standard- (*P* = .0061) and short-stem (*P* = .0055) groups at the fifth year (Table 4).

**Table 3:** Amount of bone remodeling per the Gruen zone observed at the second and fifth year.

Follow-up	Gruen zone	Standard stem			Short stem		
		Radiolucent lines	Cortical hypertrophy	Spot welds	Radiolucent lines	Cortical hypertrophy	Spot welds
Second year	1	6	0	0	7	0	2
	2	0	0	1	1	0	2
	3	0	6	15	0	32	13
	4	1	0	0	11	1	0
	5	0	16	47	2	28	36
	6	0	0	2	0	0	1
	7	0	0	0	0	0	0
Fifth year	1	2	0	1	4	1	3
	2	0	0	1	0	0	3
	3	0	9	27	0	38	19
	4	1	0	0	6	2	0
	5	0	18	59	1	31	49
	6	0	0	1	0	0	3
	7	0	0	0	0	0	0

**Table 4:** Results of Fisher's exact test performed with availability of distal bone fixation (cortical hypertrophy or spot welds in zone 3 or 5) and severity of stress shielding.

	Standard stem		Short stem	
	Second year	Fifth year	Second year	Fifth year
Mild stress shielding	50	51	68	58
Distal bone fixation +				
Mild stress shielding	38	26	27	20
Distal bone fixation −				
Severe stress shielding	9	22	5	22
Distal bone fixation +				
Severe stress shielding	3	1	0	0
Distal bone fixation −				
*P* value	0.35	0.0061^a^	0.32	0.0055^a^

+, positive; −, negative.

a Statistically significant.

Multiple regression analysis revealed a significant relationship between stress shielding and the CFI, CI, height, and stem size in the standard-stem group although the CI was the only factor that affected stress shielding in the short-stem group (Table 5).

**Table 5:** Multiple regression analysis performed to indicate factors that affect stress shielding.

Follow-up	Independent variables	Standard stem			Short stem		
		Estimated value	95% Confidence interval	*P* value	Estimated value	95% Confidence interval	*P* value
Second year	Intercept	4.0486	1.0141 to 7.0830	.0095	3.4122	1.1045 to 5.7199	.0042
	Canal flare index	0.4336	0.1754 to 0.6919	.0012^a^	0.1112	−0.0802 to 0.3025	.2516
	Cortical index	−4.4513	−7.1757 to −1.7269	.0016^a^	−3.9105	−6.2509 to −1.5700	.0013^a^
	Height	−0.0238	−0.0394 to −0.0081	.0034^a^	0.0023	−0.0119 to 0.0164	.7484
	Stem size	0.1813	0.0691 to 0.2935	.0018^a^	−0.022	−0.1165 to 0.0725	.6446
Fifth year	Intercept	3.95	0.6085 to 7.2916	.021	4.1977	1.4736 to 6.9219	.0029
	Canal flare index	0.5424	0.2580 to 0.8267	.0003^a^	0.1427	−0.0832 to 0.3686	.2128
	Cortical index	−5.6699	−8.6700 to −2.6698	.0003^a^	−3.6244	−6.3872 to −0.8615	.0107^a^
	Height	−0.0186	−0.0359 to −0.0014	.0345^a^	−0.0088	−0.0255 to 0.0079	.3000
	Stem size	0.1684	0.0449 to 0.2919	.0081^a^	0.0523	−0.0593 to 0.1638	.3547

CI, cortical index.

a Statistically significant.

One stem subsidence was recorded in the standard-stem group. All stems in both groups were considered to have bone ingrowth.

There were no significant differences in the JOA scores between the 2 groups (Table 6). The total and subsection scores of the JOA significantly improved after surgery (*P* < .0001). The prevalence of thigh pain in the standard-stem group was 2.4% at the second year and 3.5% at the fifth year. Similarly, in the short-stem group, 3.2% and 4.2% of patients expressed thigh pain at the second and fifth year, respectively.

**Table 6:** Results of clinical outcomes.

Follow-up	JOA score	Standard stem	Short stem	*P* value
Preoperative	Pain	15.6 ± 5.9	15.6 ± 5.6	.94
	ROM	13.0 ± 3.2	12.4 ± 3.4	.31
	Walking ability	9.4 ± 3.1	9.1 ± 3.6	.52
	ADL	12.7 ± 2.5	12.0 ± 3.0	.08
	Total	50.7 ± 9.6	49.0 ± 11.2	.30
Second year	Pain	38.5 ± 2.4	38.4 ± 2.7	.75
	ROM	16.2 ± 2.6	15.9 ± 2.0	.32
	Walking ability	16.5 ± 3.7	16.5 ± 3.6	.94
	ADL	17.0 ± 2.6	17.1 ± 2.4	.98
	Total	88.3 ± 8.2	87.8 ± 7.5	.66
Fifth year	Pain	38.7 ± 2.5	38.7 ± 2.7	.92
	ROM	16.4 ± 2.5	16.2 ± 2.1	.51
	Walking ability	16.9 ± 3.5	16.7 ± 3.8	.72
	ADL	17.3 ± 2.6	17.2 ± 2.5	.83
	Total	89.3 ± 8.5	88.7 ± 8.1	.66

ADL, activities of daily living; ROM, range of motion.

Dislocation was recorded in 3 patients in the standard-stem group and in 2 patients in the short-stem group. One patient in the standard-stem group required revision surgery of the polyethylene liner and head due to recurrent dislocation. The other 4 patients had no repeated dislocation. One case of postoperative fracture occurred in each group and were treated by internal fixation operation using a periprosthetic femoral fracture plate. Therefore, no patient required stem revision during follow-up.

## Discussion

The midterm clinical and radiological results with standard and short stems were favorable. No stem showed aseptic loosening, and the JOA score improved significantly. Several reports [[6], [7], [8], [9]] show the excellent survivorship of fit-and-fill stems, and the outcomes of our study regarding the short-stem variation are in keeping with the results of these reports. Unfortunately, the severity of stress shielding in the mid-term results of our study was similar to that of a previous report [10] in the Japanese population. In addition, a following report [11] by the same author mentions progressive stress shielding in the long-term results. Although these 2 reports are results of the original stem, there are concerns that standard and short stems may have the same trend of stress shielding.

The review of the multiple regression analysis results of standard stems, femoral morphology, height, and stem size showed individual effects on stress shielding. If the use of a standard stem is planned, it may be too complicated to estimate stress shielding in the future. Some reports [4,5] have shown that Asians have a smaller skeleton size, greater femoral bowing, and thinner femoral cortices. We believe that the mismatch between the Japanese femur and the standard stem leads to complications, such as increased stress shielding. In contrast, the results of using the short stem show that only the CI has a significant effect, as low CI (thin femur bone cortex) leads to severe stress shielding. In terms of predicting stress shielding, the short stem can be used easily for the Asian femur because less factors need to be considered.

There was no significant difference between the degree of stress shielding of the 2 groups although a high frequency of cortical hypertrophy was observed in zones 3 and 5 in the short-stem group. Comparing standard and short stem, the diameter of the short stem reduces faster as you go distal. Therefore, the strain in zones 3 and 5 should be greater in the short-stem group. Distal cortical hypertrophy is said to be a result of abnormal bone strain; however, its influence on outcomes is controversial [[18], [19], [20]]. In our study, distal cortical hypertrophy had no significant impact on stress shielding or thigh pain. Since there were no complications associated with this process, it is difficult to judge whether it is a positive or negative change; therefore, further research is required. At least up to the fifth year, stem length reduction has been achieved without adverse effects. The advantage of this is that for revision surgery, easier implant removal and more preserved bone stock can be expected.

Regarding the concept of a fit-and-fill stem that better matches the Asian femur, a report [21] discusses the idea of further shortened stems. Although there are few differences in the intended design, they hypothesize that even shorter stems can achieve physiological femoral loading without losing stability.

The main strength of our study is that this is a unique report comparing results between standard and shortened fit-and-fill type stems. Successful results in the Japanese population support a short-length fit-and-fill stem as a reliable implant. However, this study has several limitations. First, a relatively large number of patients (radiographs of 24% in each group, full records of 40% in the standard-stem group and 33% in the short-stem group) were lost to follow-up. This missing value can affect actual results. Fortunately, there were no revision reports or requests for primary surgery information, and it was therefore assumed that most of the patients’ implants are functioning well. Second, radiographs were analyzed by a single observer only, lacking interobserver and intraobserver differences. The accuracy and reproducibility of radiographs have been stated to be unreliable [22]. In addition, due to poor posture reproducibility, assessment of lateral radiographs was not performed. Finally, since this was a retrospective and nonrandomized study, potential bias must also be considered. For instance, surgical skills may have some improvement in the later (short stem) year.

## Conclusions

The outcomes of both standard and short fit-and-fill stems were favorable. Regarding the degree of stress shielding, there was no significant difference between the 2 stems. However, there were differences in bone remodeling and factors affecting stress shielding. Stem length reduction has been achieved without adverse effects with the Japanese femur; however, long-term follow-up is required.

## Appendix A Supplementary data

Conflict of Interest Statement for Ochi

Conflict of Interest Statement for Nozawa

Conflict of Interest Statement for Ishijima

Conflict of Interest Statement for Kato

Conflict of Interest Statement for Kim

Conflict of Interest Statement for Sakamoto
