# Supplementary material for: Comparison of the 5-Year Outcomes Between Standard and Short Fit-and-Fill Stems in Japanese Populations
Source: Arthroplast Today. 2022 Apr 25;15:108–14. doi: 10.1016/j.artd.2022.03.023 (PMC9237240; doi:10.1016/j.artd.2022.03.023)
Supplement: Conflict of Interest Statement for Ishijima [file mmc3.pdf]

INDIVIDUAL CONFLICT OF INTEREST STATEMENT

American Association of Hip and Knee Surgeons

(Adopted from the American Academy of Orthopaedic Surgeons disclosure statement)

The following form **must be filled out completely and submitted by each author (example, 6 authors, 6 forms). All items require a response. If there is no relevant disclosure for a given item, enter "*None.*"**

Comparison of the 5-year outcomes between standard and short fit-and-fill stems

Manuscript Title

1. Royalties from a company or supplier (The following conflicts were disclosed)
   None

2. Speakers bureau/paid presentations for a company or supplier (The following conflicts were disclosed)
   [1] Ono Pharmaceutical Co.Ltd., [2] Pfizer Japan Inc., [3] Taisho Pharmaceutical Holdings, [4] Kaken Pharmaceutical Co. Ltd., [5] Teijin HealthCare Co. Ltd.

3A. Paid employee for a company or supplier (The following conflicts were disclosed)
   None

3B. Paid consultant for a company or supplier (The following conflicts were disclosed)
   None

3C. Unpaid consultants for a company or supplier (The following conflicts were disclosed)
   None

4. Stock or stock options in a company or supplier (The following conflicts were disclosed)
   None

5. Research support from a company or supplier as a Principal Investigator (The following conflicts were disclosed)
   [1] Japan Medical Dynamic Marketing (JMDM) Co. Ltd., [2] Olympus Terumo Biomaterials Co. Ltd., [3] Suntory Wellness Co. Ltd., [4] CellSource Co. Ltd., [5] MATHYS Co. Ltd., [6] Stryker Co. Ltd.

6. Other financial or material support from a company or supplier (The following conflicts were disclosed)
   None

7. Royalties, financial or material support from publishers (The following conflicts were disclosed)
   None

8. Medical/Orthopaedic publications editorial/governing board (The following conflicts were disclosed)
   None

9. Board member/committee appointments for a society (The following conflicts were disclosed)
   [1] Osteoarthritis Research Society International (OARSI): Board member for Board of Directors

Each author must sign AND print or type his/her name, date and submit a separate form

In addition, one BLINDED Conflict of Interest form (no author names used) should be submitted per manuscript with all author disclosures.

Muneaki ISHIJIMA	[signature]	9/11/21
Author Name (Print or Type)	Author Signature	Date
